# Multiple choices for HIV therapy with integrase strand transfer inhibitors

**DOI:** 10.1186/1742-4690-9-110

**Published:** 2012-12-19

**Authors:** Francois Raffi, Mark A Wainberg

**Affiliations:** 1 University Hospital of Nantes, EA 4271, 1 Place Ricordeau, Nantes cedex 1 44093, France; 2 McGill University AIDS Centre, Lady Davis Institute, Jewish General Hospital, 3755 Côte-Sainte-Catherine Rd, Montreal, QC H3T 1E2, Canada

**Keywords:** Raltegravir, Elvitegravir, Dolutegravir, Integrase strand transfer inhibitors

## Abstract

Two integrases inhibitors, raltegravir and elvitegravir, have now been approved by regulatory agencies for use in the treatment of HIV-infected patients; and the approval of a third such drug, dolutegravir, is expected during 2013 on the basis of several phase 3 clinical trials. The advent of this new class of antiretroviral (ARV) medications represents a major advance in the management of HIV infection, and each of these three drugs can be expected to continue to be an important component of ARV combination regimens.

## 

The recent approval by the Food and Drug Administration (FDA) of Elvitegravir (EVG) as an anti-HIV integrase strand transfer inhibitor (INSTI) is good news for patients who will want to take a potent once-daily single tablet antiretroviral regimen (STR). INSTIs target the HIV enzyme integrase through a selective effect on strand transfer. This is a result of both the binding of the inhibitor to the specific complex between integrase and viral DNA, resulting in displacement of the reactive 3′ end of the viral DNA and chelation of the two essential magnesium ions present in the integrase active site [1]. In general, the INSTI class of antiretroviral drugs is active against multiple retroviruses, including primate and nonprimate lentiviruses, and against both HIV-1 and HIV-2 [2,3]. Furthermore, these compounds seem to be active against all subtypes of HIV-1 since the integrase enzymes of these subtypes share sequence homology and behave similarly in biochemical assays [4]. Due to the fact that EVG needs to be boosted by an additional drug, termed cobicistat, with which it has been co-formulated, the new STR will consist of four separate ingredients that also include the two potent nucleos(t)ide reverse transcriptase inhibitors (NRTIs) emtricitabine (FTC) and tenofovir (TDF). The FDA approved EVG for treatment of drug-naïve patients based on three large clinical trials, two in drug-naïve and one in drug-experienced patients [5-7]. The efficacy and safety of the four drugs in the new formulation, now termed Stribild®, was evaluated in a randomized double-blind double dummy design against a triple drug STR termed Atripla® that includes both FTC and TDF as well as the non-nucleoside reverse transcriptase inhibitor (NNRTI) efavirenz (EFV) in a trial of 700 drug-naïve patients [5]. In a second trial of 700 drug-naïve patients, the same four drug STR was evaluated against a triple combination that includes both FTC and TDF as well as the ritonavir-boosted protease inhibitor (PI) atazanavir (ATV/r) [6]. In these two trials, the new EVG-containing regimen was documented to be non-inferior to Atripla® and to TDF/FTC plus ATV/r in terms of the proportion of previously drug-naïve patients demonstrating viral load suppression to below 50 copies RNA/ml after 48 weeks of treatment. The new regimen is considered by many to have potential advantages compared with Atripla® because of well-documented EFV-related toxicities (central nervous system (CNS) and cutaneous) and the fact that some patients may have been infected by viruses that contained the K103N mutation or other mutations that confer resistance against EFV. Even though any patient possessing these mutations on the basis of standard genotyping prior to initiation of therapy would be ineligible to receive either EFV or Atripla®, the fact is that some patients may possess minority variants of these mutations that can only be detected by more sensitive techniques, not available in routine practice, and that such individuals may be compromised in terms of ability to respond to an EFV-based regimen [8].

At the same time, it must be recognized that one other INSTI is currently available for treatment and that other members of this family of compounds are now in late stage clinical development and may be approved for therapy in the near future. The drug that has already been approved is raltegravir (RAL), and it has the advantage of being extremely potent as well as safe and very well tolerated over more than five years of clinical experience [9]. Moreover, the Startmrk trial has reported long term efficacy data of 563 previously treatment-naive individuals over five years of therapy, and the results reveal that RAL is virologically superior to EFV over this time and better tolerated when either drug is used in combination with FTC and TDF, despite the fact that EFV can be dosed once-daily while RAL has only been approved for use as a twice-daily drug [10]. The Startmrk trial was a randomized double-blind study in which RAL was dosed twice daily while patients on the EFV arm of the study received active EFV as part of their night-time regimen and a placebo in the morning at the same time that patients in the RAL arm received active drug [10]. In view of the fact that EFV possesses a far longer plasma half-life than RAL, it can therefore be further argued that any degree of non-adherence in the Startmrk trial should have favoured the EFV arm. Thus, the demonstrated superiority of RAL over EFV is even more impressive than the data of the intent-to-treat analysis would seem to indicate (proportion with HIV RNA < 50 c/ml at week 240 was 71% vs. 61% for RAL and EFV, respectively (95% CI : 1.7-17.3)) [9]. Of note there was a low level of resistance among patients with virologic failure; after 240 weeks of follow-up, only 7 patients in the RAL group possessed resistance mutations at time of virologic failure. There was also a benefit of RAL over EFV in regard to lipid profile [9].

It is worth noting that RAL was approved by regulatory agencies as a twice daily drug on the basis of the Startmrk clinical trial. However, attempts were made to demonstrate that RAL might be used on a once-daily basis if dosed at 800mg once-daily (qd) as opposed to the twice-daily (bid) dosing schedule of 400 mg that is recommended [11]. Although the results of the clinical trial on this topic, termed Qdmrk, performed in 670 drug-naïve individuals, showed that qd dosing with RAL in combination with TDF/FTC did not meet the 10% criterion of non-inferiority in comparison to the bid use of RAL, the fact is that the qd use of RAL proved to be effective at suppression of viral load to <50 copies viral RNA/ml in more than 83% of subjects over a period of 48 weeks [12]. Moreover, the results further indicated that qd dosing with RAL was as effective as bid RAL in almost all patients who initiated therapy with HIV RNA <100,000 copies/ml at baseline, suggesting as well that RAL may be a forgiving drug in regard to occasional non-adherence [12,13]. One of the reasons for the superiority of RAL over EFV in the Startmrk trial may, therefore, be that occasionally missed doses of RAL as part of a bid regimen may not impact significantly on treatment outcome, and, as well, that RAL is both more potent and less toxic than EFV. Of course, the fact that RAL does not need to be boosted by a pharmacological enhancer such as ritonavir or cobicistat is another major advantage.

Recent clinical findings suggest that a new second generation INSTI termed dolutegravir (DTG) is also superior to EFV over 48 weeks of therapy, when both drugs are used in combination with a fixed dose of once-daily NRTIs, abacavir/lamivudine or TDF/FTC, respectively [14]. However, a second randomized double-blind double dummy study showed that once daily DTG was not superior but rather non-inferior to twice daily RAL, despite the potential advantage of being used on a qd basis due to a long plasma half-life and an ability to bind to the HIV integrase enzyme for a more protracted period than either RAL or EVG [15]. DTG also has the advantage of not requiring boosting by a pharmacological enhancer, and it is likely that DTG will be approved by regulatory agencies during 2013, giving rise to a situation in which three different INSTIs will be available for treatment of HIV disease. DTG will represent a further important addition to the antiretroviral armamentarium of drugs and has also the advantage of having a higher genetic barrier to resistance than the two other INSTIs (Table 1, [3]).

**Table 1:** Major characteristics of the 3 INSTIs

**Characteristic**	**RAL**	**EVG/cobi**	**DTG**
Dosing	400 mg bid	150/150 mg qd	50 mg qd in INSTI-naive and 50 mg bid in INSTI-experienced patients
STR	No	Yes (TDF/FTC/EVG/cobi)	Together with abacavir(ABC) and 3TC
To be taken with food	No	Yes	No
In vitro activity*	33 nM (IC_95_)	45 ng/mL (IC_95_)	0.064 μg/mL (0.15 μM) (IC_90_)
Protein binding	83%	98 %	99.3%
Terminal half-life	9 h	12.9 h/3.5 h	15 h
Drug-drug interactions	with inducers of UGT1A1 (rifampin)	Presence of a strong CYP3A inhibitor such as cobicistat creates the potentialfor an increase in systemic exposure of CYP3A substrates	with inducers of UGT1A1 (rifampin)
Interaction with proton pump inhibitors and antacids	No	No	No
Major resistance mutations [summarized in 3]	E92Q	T66I/A/K	None (accumulation of multiple mutations required to confer resistance)
	Y143C/H/R	E92Q/G	
	Q148H/K/R	T97A	
	N155H	S147G	
		Q148H/R/K	
		N155H	

*RAL* = raltegravir ; *EVG/cobi* = elvitegravir/cobicistat ; *DTG* = dolutegravir ; *bid* = twice-daily ; *qd* = once-daily ; *TDF/FTC* = tenofovir/emtricitabine ; *ABC/3TC* = abacavir/lamivudine.

* in vitro protein-adjusted inhibitory concentration.

Of note, comparison of the different phase 3 studies of the three INSTIs is difficult. For example, median baseline plasma viral load was significantly higher in the Startmrk (5.1 log_10_ c/ml) than in the Stribild® studies (4.75 and 4.88 log_10_ c/ml) and the DTG studies (4.52 and 4.67 log_10_ c/ml) [5,6,10,14,15]. This reinforces the clinical potency and efficacy of RAL in mediating virologic suppression to levels below detectability after 48 weeks of therapy, since virologic suppression is more difficult to achieve in patients who present with high initial plasma viral loads. One of the differences between the three INSTIs is related to inhibition of renal tubular secretion of creatinine by both dolutegravir and cobicistat which leads to a rapid and sustained increase of serum creatinine, although glomerular filtration is not affected [5,6,14,15]. Furthermore, some cases of discontinuations for renal toxicity with proximal tubulopathy occurred in one of the phase 3 trials of TDF/FTC/EVG/cobicistat (5) and Stribild® is contra-indicated in patients with estimated creatinine clearance below 70 mL per minute.

Table 1 summarizes the main characteristics of the three INSTIs. RAL is classified by the FDA as a category C drug for use during pregnancy, and the STR of TDF/FTC/EVG/cobicistat as category B, while information on this topic is not yet available for dolutegravir. Based on package inserts, RAL is not recommended for use during pregnancy, and TDF/FTC/EVG/cobicistat should be used during pregnancy only if the potential benefit justifies the potential risk to the fetus. RAL has been recently approved by the FDA for use in children and adolescents aged 2–18. Chewable pills are available for children aged 2 to 11, while the safety and effectiveness of TDF/FTC/EVG/cobicistat and DTG in pediatric patients less than 18 years have not been established. A study evaluating RAL in pregnancy is ongoing (clinicaltrials.gov NCT01618305).

Thus, we are privileged to have multiple options in regard to the use of various INSTIs for therapy of HIV-infected individuals. EVG is the newest INSTI to be approved, and the promise of DTG for the future therapy of HIV disease is exciting. Resistance against both RAL and EVG have been reported on the basis of both clinical failures (fewer with RAL than EVG) and tissue culture drug selection studies, and it is clear from this work that the most frequent mutations in the HIV integrase gene that confer resistance against RAL also confer resistance against EVG, making it highly improbable that these two molecules might be used to salvage one another in the event of drug resistance [3]. Indeed, EVG can overcome only one of the three RAL-resistance pathways (Y143). On the other hand, DTG has demonstrated clinical activity at double the standard dose of 50 mg bid in patients harboring viruses resistant to RAL and/or EVG [3]. However, an accumulation of the major RAL- or EVG-resistance mutations may also diminish the likelihood of long-term clinical success with DTG. At the same time, RAL, the first approved member of the INSTI family of drugs, remains the only integrase inhibitor to have proven itself over more than five years of clinical experience as a fully safe and effective compound, without significant drug-drug interactions, that is superior to EFV. Although RAL is only recommended for twice daily dosing, an analysis of the Qdmrk study and other recent studies in which virologically suppressed patients were switched to other regimens suggests that a once daily dose of 800 mg RAL could represent an option for first-line therapy in some patients presenting with HIV RNA < 100,000 c/ml or in virologically suppressed patients who wish to change regimens from a prior first-line boosted protease inhibitor containing regimen. Such qd dosing of RAL should be further evaluated in randomized settings

## Conclusions

Each of RAL, EVG, and DTG will continue to be important components of combination anti-HIV therapy over many years. Although there are more long-term efficacy and safety data now available on RAL than on the other two drugs, the use of the other options in first-line therapy is also compelling. Due to its more favorable resistance profile, DTG will probably be the only member of the INSTI family of drugs that will be useful in both first-line therapy as well as in subsequent HIV INSTI-based treatment.

## Competing interest

Drs Raffi and Wainberg have received research funding and/or consultancy honoraria from Gilead Sciences, Merck Inc., ViiV Healthcare and Bristol-Myers Squibb Inc.

## Authors’ contributions

Both FR and MAW conceived of the idea for this Viewpoint, and both authors contributed to its writing. Both authors read and approved the final manuscript.
